# Real-world evidence on Kovaltry (81-8973) in children with moderate or severe hemophilia A in Europe: a nested cohort analysis

**DOI:** 10.1186/s13023-021-01676-w

**Published:** 2021-01-15

**Authors:** Jamie O’Hara, Ceri Hirst, Jose Francisco Cabre Marquez, Tom Burke

**Affiliations:** 1 Department of Health and Social Care, University of Chester, Chester, UK; 2 HCD Economics, Daresbury, UK; 3 Real World Evidence, Bayer, Basel, Switzerland; 4 Global Medical Affairs, Bayer, Mexico City, Mexico

**Keywords:** Hemophilia A, Kovaltry, Real world evidence, Factor utilization, Clinical burden

## Abstract

**Background:**

Untreated hemophilia A patients may experience recurrent bleeding events leading to debilitating joint damages. While RCT and pharmacokinetic data support the value of Kovaltry [an unmodified full-length recombinant factor VIII (FVIII) product], real world evidence in children is lacking. This report describes a descriptive and multivariate analysis of the effectiveness of Kovaltry in children with hemophilia A in the real-world setting, using data from medical chart abstraction and cross-sectional surveys of physicians, patients, and caregivers.

**Results:**

Male patients aged < 18 years with moderate or severe hemophilia A, residing in five European countries and treated with FVIII were studied. The co-primary endpoints were the annualized bleeding rate (ABR) and the annual FVIII utilization rate. Twenty nine patients treated with Kovaltry were included, of whom 93% had severe disease and 75% were on continuous prophylactic treatment. The mean ABR was 2.66 ± 2.06, with rates decreasing with age. The children received on average 2.45 infusions per week, consistent across age groups (median 3; range 1–3). There were no reports of inhibitor development or adverse events in the study (AEs), and all patients were satisfied or very satisfied with the treatment. An exploratory multivariate analysis suggests no significant difference in ABR or units utilized between Kovaltry and some extended half life products in children with severe hemophilia A, though characteristics of these patient cohorts were markedly different.

**Conclusion:**

This analysis demonstrates the effectiveness and safety of Kovaltry in a pan-European pediatric population with severe hemophilia A.

## Introduction

Hemophilia A is a rare X-linked genetic disorder characterized by deficiency of coagulation factor VIII (FVIII) [1–4]. Individuals with severe hemophilia have FVIII levels less than 1% of that expected in a healthy person [5, 6]. Such patients, if not optimally treated, encounter recurrent bleeding episodes causing cumulative damage and commonly arthropathy [7, 8].

In Western European countries, around 90% of children with severe hemophilia A now have access to specialist care teams and prophylactic treatment with replacement FVIII, which is proven to reduce bleeding events and resultant joint damage [9–12]. Two main types of replacement FVIII are now available; standard half-life products (SHL) with half-life of around 8–12 h [13], and more recently, so-called extended half life products (EHL) [14, 15]. Two EHL products were approved in Europe at the time of this study: Elocta (Sobi) in 2015 [14] and Adynovate (Takeda) in 2018 [15] only for people over the age of 12 years. There is little evidence on the relative clinical effectiveness of SHL and EHL products in the real world setting and particularly in children.

Kovaltry is an unmodified full-length recombinant SHL FVIII product, yet pharmacokinetic analyses have shown an increased half-life for Kovaltry when compared with traditional FVIII products in adult hemophilia A patients, and consistency in terminal half-life for children across all age groups [16–19]. Currently there is an evidence gap in translation of the improved PK profile to clinical benefits in children: real-world utilization, safety, and effectiveness, particularly in comparison with EHL products.

The primary objective of this study was to describe the effectiveness of Kovaltry in children with hemophilia A in the real-world setting.

## Methods

A nested cohort analysis was conducted utilizing data as captured in the Cost of Haemophilia in Europe: a Socioeconomic Survey (CHESS) Pediatrics study [20, 23]. Clinical- and patient-reported data were obtained between December 2017 and April 2018 via medical chart abstraction, including 12 months of retrospective clinical data and cross-sectional surveys from physicians and patients/caregivers. Males aged less than 18 years who had moderate or severe hemophilia A; resided in France, Germany, Italy, Spain, or the United Kingdom (UK); and who were treated with FVIII were included in analyses. The co-primary endpoints were the annualized bleeding rate (ABR) as reported by the physicians in the survey and the annual Kovaltry utilization. Standard descriptive analyses were used to describe baseline characteristics and outcomes associated with the included patients [21].

In an exploratory analysis, we compared the characteristics of patients treated with Kovaltry and EHL products in this real world setting. The impact of covariates on outcome variables was assessed using a generalized linear model (GLM) procedure with stepwise model selection to control covariates and minimize the confounding effect. ABR was described and included as a covariate in the multivariate analysis of treatment utilization. Conversely, treatment utilization was included in the ABR model. Sensitivity analysis excluded patients having a treatment dose considered by the steering committee to be unfeasible: < 2.5 IU/kg/week and > 200 IU/kg/week. Statistical analyses were performed using SAS® software Version 9.4.

The sample size was driven by the full number of eligible patients available in the dataset, with no further restrictions. The rarity of haemophilia A often limits the sample size available for research, particularly in children, and although CHESS Pediatrics provides one of the largest datasets in this population, the statistical power is limited. As such, all analyses are considered descriptive and exploratory.

## Results

In the primary descriptive analysis of Kovatry, 29 children were included (Table 1). Nearly all patients had severe hemophilia (93.1% *n* = 27). Most were on continuous prophylaxis at the initiation of the study (74.9%; *n* = 22). Target joints at the study initiation were rare (11%).

**Table 1:** Demographic characteristics of patients receiving Kovaltry and EHL

Patient characteristics	All Kovaltry (n = 29)	Prophylactic Kovaltry (*n* = 22)	Prophylactic EHL (*n* = 60)
Age group *n* (%)			
0–5 years	5 (17.2)	3 (13.6)	3 (5.0)
6–11 years	15 (51.7)	11 (50.0)	15 (25.0)
12–17 years	9 (31.0)	8 (36.4)	42 (70.0)
Body mass index groups			
Underweight < 18.5	10 (34.5)	8 (36.4)	9 (15.0)
Normal 18.5–24.9	9 (31.0)	8 (36.4)	40 (66.7)
Overweight 25–29.9	5 (17.2)	2 (9.1)	6 (10.0)
Obese 30+	4 (13.8)	3 (13.6)	4 (6.7)
Missing	1 (3.4)	1 (4.5)	1 (1.7)
Severity			
Moderate (1–5 IU/dL)	2 (6.9)	2 (9.1)	12 (20.0)
Severe (< 1 IU/dL)	27 (93.1)	20 (90.9)	48 (80.0)
History of inhibitors			
0 Never	23 (79.3)	17 (77.3)	46 (76.7)
1 Once	6 (20.7)	5 (22.7)	10 (16.7)
More than once—recurrence/relapse	0 (0)	0 (0)	4 (6.70)
Treatment regimen at initiation			
Continuous prophylaxis	22 (75.9)	17 (77.3)	24 (40.0)
Episodic—on demand—treatment	6 (20.7)	3 (13.6)	32 (53.3)
Intermittent—periodic—prophylaxis	1 (3.4)	2 (9.1)	4 (6.7)
Target joints			
No	26 (89.7)	20 (90.9)	49 (81.7)
Yes	3 (10.3)	2 (9.1)	11 (18.3)
Chronically damaged joints			
No	26 (89.1)	21 (95.5)	47 (78.3)
Yes	3 (10.3)	1 (4.5)	13 (21.7)
Treatment adherence			
Fully adherent: missing < 15% of infusions	25 (86.2)	18 (81.8)	51 (85.0)
Sub-optimally adherent: missing 15–25% of infusions	3 (10.3)	3 (13.6)	7 (11.7)
Non-adherent: missing > 25% of infusions	0	0	0 (0)
Not applicable	1 (3.4)	1 (4.5)	2 (3.30)

The mean annual bleeding rate in patients treated with Kovaltry was 2.66 (SD ± 2.06). The data demonstrated a reduction in rates of bleeding according to age categories, from 4 ± 2.92 in the youngest children aged 0–5 years, 2.67 ± 1.84 in those aged 6–11 years, and in the sub group aged 12–17 years 1.89 ± 1.69. Around 20% of the patients who were receiving Kovaltry were free of bleeds; (*n* = 6). Within the 22 patients receiving Kovaltry prophylactically, the mean ABR was 2.18, and after excluding one patient with an unfeasible dosage was 1.90. On average, these patients were prescribed 2.45 infusions per week (median 3; range 1–3).

There were no reports of inhibitor development or any adverse events (AEs) with Kovaltry. Most patients (86.2%; *n* = 25) were considered by their physician to be adherent to their prescribed treatment regimen (missing < 15% of infusions). All patients who responded to the linked patient survey were satisfied with the treatment, and 60% were very satisfied.

### Exploratory analysis

In the exploratory analysis, 82 patients were included (22 Kovaltry and 60 EHL). Characteristics of patients prescribed Kovaltry and EHL differed considerably. Patients in the Kovaltry cohort were younger in age, had more severe disease, and started prophylactic treatment at a younger age. Patients in the EHL cohort were more likely to have had recurrent inhibitors, target joints, and damaged joints. The physician-reported adherence rate was similar between both groups (Table 1).

**Fig. 1:**
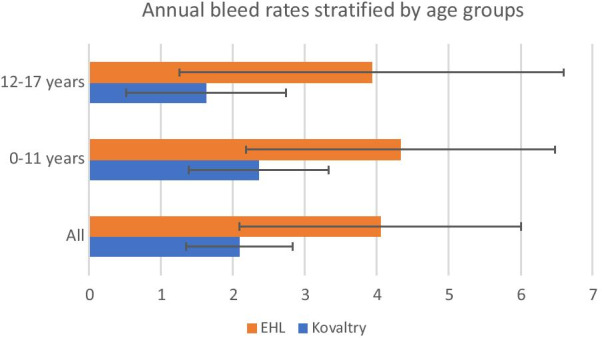
Annual bleed rates stratified by age group

The mean ABR in the Kovaltry cohort was lower than with EHL (ABR 1.90 ± 1.58 vs 4.14 ± 7.88 respectively, *P* = 0.04). After statistical adjustment for differences in baseline patient characteristics (age, severity, utilization, presence of target or chronic joints), the difference was not statistically significant (ABR 2.18 ± 1.55 vs 4.06 ± 0.90 respectively (*P* = 0.31). ABR remained lower in Kovaltry treated patients across all age groups (0–11 years 2.36 vs. 4.33 and 12–17 years 1.63 vs. 3.93 for Kovaltry and EHL, respectively) (Fig. 1), and a higher proportion of patients in the Kovaltry cohort had zero annual bleed rates when compared with EHL (27.3% vs. 10%).

The mean weekly infusion frequency was comparable between the Kovaltry and the EHL group (2.43 vs. 2.28) and was similar across all age groups.

The calculated median factor utilization was lower in the Kovaltry group than in the EHL group (weekly 80.1 IU/kg vs 89.9 IU/kg; *P* = 0.43). When adjusting for differences in baseline characteristics in multivariate analyses, there was a numerical, but not statistically significant, difference between Kovaltry and EHL cohorts, (74.84 IU/kg vs. 88.83 IU/kg; *P* = 0.23).

## Discussion

While randomized clinical trials (RCTs) are recognized as the gold standard for evaluating the efficacy of new products, in a real-world setting both the characteristics of patients and the way in which products are used can differ from the trial setting [22, 23]. Therefore, this RWE in pediatric patients across Europe adds to the scientific knowledge of hemophilia A treatment.

This current analysis supports the strong profile of Kovaltry, with a low mean annual bleeding rate of 2.18, ranging from 2.67 to 1.89 from the youngest to the oldest patients, in line with clinical and pharmacokinetic expectations [17–20]. 100% patient satisfaction and an average of 2.45 infusions per week were reported. No adverse events or inhibitor development was reported, in line with previous research [12–14].

The exploratory analysis demonstrates that, at the time of the analysis, children prescribed EHL products had markedly different characteristics from those prescribed Kovaltry, being typically older and more likely to have had recurrent inhibitors, target joints, and damaged joints. The noted non-significant differences in ABR and utilization must be interpreted cautiously in light of the low sample size, due to the disease rarity, limited available EHLs at the time of the study, restriction of use to only patients aged above 12 years for Adynovi, and potential for residual confounding factors. Full clinical details of joint involvement, such as imaging, were also not available in the study dataset.

This study highlights the importance of observational data to better understand the utilization of new FVIII products in the real world, particularly how this may impact patient management and outcomes. It also indicates that further research may be warranted to understand the relative value of FVIII products in children.

## Data Availability

All the data was collected from the CHESS Pediatrics study and is available with the corresponding author upon reasonable request.

## Abbreviations

ABR: Annualized bleeding rate
AEs: Adverse events
AUC: Area under the plasma drug concentration–time curve
BMI: Body mass index
CHESS: Cost of Haemophilia in Europe: a Socioeconomic Survey
CIs: Confidence intervals
EHL: Extended half-life
EMA: European Medicines Agency
FVIII: Factor VIII
GLM: Generalized linear model
*n*/*N*: Number
PK: Pharmacokinetic
RCTs: Randomized clinical trials
RWE: Real-world evidence
SD: Standard deviation
SE: Standard error
SHL: Standard half-life
UK: United Kingdom
